# Improvement of Xylose Fermentation Ability under Heat and Acid Co-Stress in *Saccharomyces cerevisiae* Using Genome Shuffling Technique

**DOI:** 10.3389/fbioe.2017.00081

**Published:** 2017-12-20

**Authors:** Kentaro Inokuma, Ryo Iwamoto, Takahiro Bamba, Tomohisa Hasunuma, Akihiko Kondo

**Affiliations:** ^1^Graduate School of Science, Technology and Innovation, Kobe University, Kobe, Japan; ^2^Department of Chemical Science and Engineering, Graduate School of Engineering, Kobe University, Kobe, Japan; ^3^Biomass Engineering Program, RIKEN Center for Sustainable Resource Science (CSRS), Yokohama, Japan

**Keywords:** *Saccharomyces cerevisiae*, xylose, ethanol production, thermotolerance, acid tolerance, genome shuffling, transcriptome analysis

## Abstract

Xylose-assimilating yeasts with tolerance to both fermentation inhibitors (such as weak organic acids) and high temperature are required for cost-effective simultaneous saccharification and cofermentation (SSCF) of lignocellulosic materials. Here, we demonstrate the construction of a novel xylose-utilizing *Saccharomyces cerevisiae* strain with improved fermentation ability under heat and acid co-stress using the drug resistance marker-aided genome shuffling technique. The mutagenized genome pools derived from xylose-utilizing diploid yeasts with thermotolerance or acid tolerance were shuffled by sporulation and mating. The shuffled strains were then subjected to screening under co-stress conditions of heat and acids, and the hybrid strain Hyb-8 was isolated. The hybrid strain displayed enhanced xylose fermentation ability in comparison to both parental strains under co-stress conditions of heat and acids. Hyb-8 consumed 33.1 ± 0.6 g/L xylose and produced 11.1 ± 0.4 g/L ethanol after 72 h of fermentation at 38°C with 20 mM acetic acid and 15 mM formic acid. We also performed transcriptomic analysis of the hybrid strain and its parental strains to screen for key genes for multiple stress tolerances. We found that 13 genes, including 5 associated with cellular transition metal ion homeostasis, were significantly upregulated in Hyb-8 compared to levels in both parental strains under co-stress conditions. The hybrid strain Hyb-8 has strong potential for cost-effective SSCF of lignocellulosic materials. Moreover, the transcriptome data gathered in this study will be useful for understanding the mechanisms of multiple tolerance to high temperature and acids in yeast and facilitate the development of robust yeast strains for SSCF.

## Introduction

Lignocellulosic materials such as agricultural residues and forestry wastes contain large amounts of polysaccharides (cellulose and hemicellulose). These polysaccharides have attracted a lot of attention as feedstocks for second-generation bioethanol production (Hasunuma and Kondo, 2012). However, due to the rigid structures of lignocellulosic materials, pretreatment and hydrolysis by an enzyme complex are required to hydrolyze these polysaccharides to fermentable sugars such as glucose and xylose (Percival Zhang et al., 2006; da Costa Sousa et al., 2009; Hendriks and Zeeman, 2009; Alvira et al., 2010). Because of these processes, the bioconversion of these materials to ethanol is more costly than that of conventional sugar sources such as corn starch and cane juice (Cardona and Sáncheza, 2007; Jin et al., 2012). Therefore, simultaneous saccharification and cofermentation (SSCF), which combines enzymatic hydrolysis of the polysaccharides and fermentation into a single process, has great potential for the cost-effective ethanol production from lignocellulosic materials (Hasunuma and Kondo, 2012). Combining these two process steps reduces capital costs, processing time, contamination risk, and the sugar inhibition of enzymatic hydrolysis (Taherzadeh and Karimi, 2007).

*Saccharomyces cerevisiae* is the most frequently used microorganism for industrial ethanol production due to its high fermentation capacity and ethanol tolerance (Olsson and Hahn-Hägerdal, 1993; Lau et al., 2010). Although native *S. cerevisiae* is not capable of utilizing xylose as a carbon source, xylose-assimilating *S. cerevisiae* strains have been developed in the past few decades by overexpressing genes encoding the enzymes required for the assimilation of xylose (Hahn-Hägerdal et al., 2007; Nevoigt, 2008; Van Vleet and Jeffries, 2009). However, there remain some barriers to the use of *S. cerevisiae* in the SSCF of lignocellulosic materials.

During the fermentation process, yeasts encounter a variety of harmful compounds (such as weak organic acids, furan derivatives, and phenolics) generated during biomass pretreatment. These compounds can inhibit the cell growth, metabolism, and ethanol yield of *S. cerevisiae*, particularly in the presence of ethanol (Klinke et al., 2004; van Maris et al., 2006; Almeida et al., 2007). The discrepancy between the optimum temperature of hydrolytic enzymes and that of *S. cerevisiae* is also a barrier to SSCF. Typical *S. cerevisiae* requires a fermentation temperature between 30 and 35°C to maximize ethanol production, and higher temperatures inhibit both growth and fermentation (D’Amore et al., 1989). In contrast, the *Trichoderma* cellulolytic and hemicellulolytic enzymes most commonly used have an optimum temperature of around 50°C (Taherzadeh and Karimi, 2007). Therefore, SSCF using *S. cerevisiae* is often conducted at around 37–40°C to compromise between these optimal temperatures (Olofsson et al., 2008). Such temperature limitations result in a consequent decrease in ethanol production. Therefore, xylose-utilizing *S. cerevisiae* strains with tolerance to both fermentation inhibitors and high temperature are required for cost-effective SSCF of lignocellulosic materials. Many researchers have reported improvements in these yeast tolerances using techniques such as genetic engineering, adaptation, ultraviolet (UV) and chemical mutagenesis, and protoplast fusion (Steensels et al., 2014). These methods have made a lot of progress in improving the tolerances of industrial yeasts for several decades.

In the last decade, genome shuffling has attracted attention as powerful approach for enhancing genetically complex phenotypes, including stress tolerance (Biot-Pelletier and Martin, 2014). In this method, first, a population with genetic diversity is created by mutation induction in a parent strain using chemicals or UV irradiation. Second, the genomes of the mutagenized pool are shuffled by asexual (protoplast fusion) or sexual (sporulation and mating) hybridization. Finally, a library of the shuffled strains is subjected to screening in order to identify desirable strains. Genome shuffling is a particularly useful technique to generate interspecific or interstrain hybrids (Kunicka-Styczyńska and Rajkowska, 2011). Novel combinations of beneficial traits of parental strains can be generated and undesirable mutations can be removed without the acquisition of genome sequence information (Petri and Schmidt-Dannert, 2004). Moreover, genome shuffling can be combined with genetic engineering (Wang et al., 2012). Genome shuffling has yielded yeast strains with improved acetic acid tolerance (Wei et al., 2008; Zheng et al., 2011a), thermotolerance (Shi et al., 2009), ethanol tolerance (Hou, 2009), and multiple tolerances to these stresses (Zheng et al., 2011b; Lu et al., 2012).

In the present study, genome shuffling was used to improve the fermentation performance of xylose-assimilating *S. cerevisiae* under heat and acid co-stress. We previously transformed industrial diploid *S. cerevisiae* strains exhibiting thermotolerance (Sun049) and acid tolerance (Sun224) with a plasmid harboring the xylose-assimilating genes xylose reductase (*XYL1*) and xylitol dehydrogenase (*XYL2*) from *Scheffersomyces stipitis* and xylulokinase (*XKS1*) from *S. cerevisiae* and obtained the xylose-assimilating *S. cerevisiae* strains Sun049T and Sun224T, respectively (Ismail et al., 2013). These strains were used as the parental strains for genome shuffling in this study. First, different drug resistance markers were integrated into the genomes of these parental strains to eliminate unmated cells by selecting for double resistance (Zheng et al., 2011a). Genetically diverse populations of these strains were generated by mutation induction using UV irradiation, and the genomes of the mutagenized pool were shuffled by sporulation and mating. The shuffled strains were then subjected to screening under the co-stress conditions of heat and acids, and a hybrid strain with desirable traits was isolated. Subsequently, the fermentation performance of the hybrid strain was compared to that of its parental strains under different stress conditions. Finally, we performed transcriptomic analysis of the hybrid strain and its parental strains to screen for key genes involved in the fermentation performance under the co-stress conditions of heat and acids. To our knowledge, this is the first report of the application of genome shuffling to improve the fermentation performance of xylose-assimilating *S. cerevisiae* under the co-stress conditions of heat and acids.

## Materials and Methods

### Strains and Media

*Escherichia coli* strain NovaBlue (Novagen Inc., Madison, WI, USA) was used as the host for recombinant DNA manipulation. *E. coli* medium was prepared as described (Inokuma et al., 2016).

The characteristics of all yeast strains used in this study are shown in Table 1. Industrial diploid *S. cerevisiae* strains with thermotolerance (Sun049) and acid tolerance (Sun224) were obtained from Suntory Limited (Tokyo, Japan). Xylose-assimilating yeasts Sun049T and Sun224T were constructed from Sun049 and Sun224, respectively, by introducing a plasmid harboring the xylose-assimilating genes xylose reductase (*XYL1*) and xylitol dehydrogenase (*XYL2*) from *S. stipitis* and xylulokinase (*XKS1*) from *S. cerevisiae*, as well as a clonNAT resistance cassette in a previous study (Ismail et al., 2013). These strains were used as the starting strains. Yeast extract peptone dextrose (YPD) medium [10 g/L yeast extract, 20 g/L Bacto-peptone (Difco Laboratories, Detroit, MI, USA), and 20 g/L glucose] supplemented with appropriate antibiotic drugs was used to screen, grow, and maintain *S. cerevisiae* strains. Yeast extract peptone xylose (YPX) medium (10 g/L yeast extract, 20 g/L Bacto-peptone, and 50 g/L xylose) was also used for the fermentation tests.

**Table 1:** Characteristics of *Saccharomyces cerevisiae* strains and plasmids used in this study.

Name	Relevant genotype	Source
**Strains**		
Sun049T	*MAT*α*/****a***	Ismail et al. (2013)
Sun224T	*MAT*α*/****a***	Ismail et al. (2013)
Sun049T-Z	*MAT*α*/****a*** *HO/*Δ*ho::loxP-Sh Ble-loxP*	This study
Sun224T-K	*MAT*α*/****a*** *HO/*Δ*ho::loxP-KanMX-loxP*	This study
Hyb-1–8	Hybrid strains of Sun049T-Z and Sun224T-K	This study
**Plasmids**		
pUG6	Cloning vector with *loxP-KanMX-loxP* cassette	EUROSCARF
pTEF1/Zeo	Cloning vector with zeocin resistance gene (*Sh Ble*)	Thermo Fisher Scientific
pUG-Zeocin	Cloning vector with *loxP-Sh Ble-loxP* cassette	This study

### Plasmid Construction and Yeast Transformation

The plasmids used in this study are listed in Table 1. The plasmid pUG6 (Güldener et al., 1996) harboring the *loxP-kanMX-loxP* module was purchased from EUROSCARF (Bad Homburg, Germany). In order to construct a plasmid with the zeocin resistance gene (*Sh ble*), the *kanMX* cassette on pUG6 was replaced with the *Sh ble* cassette as follows: the *Bam*HI-*Xho*I DNA fragment encoding the *Sh ble* cassette was obtained from plasmid pTEF1/Zeo (Thermo Fisher Scientific Inc., Waltham, MA, USA). This fragment was inserted into the *Bgl*II and *Xho*I sites of pUG6. The resulting plasmid was designated pUG6-Zeocin.

For integration of the drug resistance markers into the *HO* gene locus of the chromosomal DNA by homologous recombination, 45 bp of sequence upstream and downstream of the *S. cerevisiae HO* gene were cloned to the left and right of the *loxP-kanMX-loxP* and *loxP-Sh ble-loxP* modules by PCR using the primers dHO-F: 5′-CATATCCTCATAAGCAGCAATCAATTCTATCTATACTTTAAAATGCTTCGTACGCTGCAG-3′ and dHO-R: 5′-TTACTTTTATTACATACAACTTTTTAAACTAATATACACATTTTAGCCACTAGTGGATCT-3′, respectively. Plasmids pUG6 and pUG6-Zeocin were used as PCR templates to generate the disruption cassettes. The amplified *loxP-Sh ble-loxP* and *loxP-kanMX-loxP* modules were then transformed into Sun049T and Sun224T, respectively, using the lithium acetate method (Chen et al., 1992) and integrated into the *HO* locus of the chromosomal DNA by homologous recombination. Transformants of Sun049T and Sun224T were selected on YPD containing 200 µg/mL zeocin and 300 µg/mL G418, and the resulting transformants were named Sun049T-Z and Sun224T-K, respectively. The integration of each module into the *HO* locus was verified by diagnostic PCR (colony PCR) using primers targeted to sequences upstream (HO-F: 5′-ACCCACTAGTACTACCATTG-3′) and downstream (HO-R: 5′-GTTAAGACTGCATTCATCACT-3′) of this locus (data not shown).

### UV Treatment

Sun049T-Z and Sun224T-K were cultivated in 5 mL YPD medium containing 50 µg/mL clonNAT (Werner Bioagents, Jena, Germany) at 30°C to an optical density at 600 nm (OD_600_) of 1.0. The culture medium was transferred to a 10-mL flask and then exposed to a germicidal lamp (GL15, TOSHIBA, Tokyo, Japan) for 2 min at a distance of 30 cm with mild agitation. The treated cells were inoculated into 200 mL YPD medium containing 50 µg/mL clonNAT and cultivated at 30°C to an OD_600_ of 1.0. The cells were harvested by centrifugation at 3,000 × *g* for 5 min. The resulting cells were washed three times with distilled water and used for sporulation.

### Sporulation and Spore Purification

Sporulation and spore purification were performed according to the procedure described by Hou (2009) with a minor modification in which brief sonication for purified spore suspensions was skipped.

### Spore Mating

The spore suspensions (100 µL) derived from Sun049T-Z and Sun224T-K were mixed in 5 mL YPD with 50 µg/mL clonNAT and incubated at 200 rpm and 30°C for 48 h. The cells and spores were harvested by centrifugation at 3,000 × *g* for 5 min; inoculated in 5 mL YPD medium with 50 µg/mL clonNAT, 200 µg/mL zeocin, and 300 µg/mL G418 at an initial OD_600_ of 0.1; and incubated at 200 rpm and 30°C for 24 h. The harvest, inoculation, and incubation were repeated once again to obtain a library of Sun049T-Z and Sun224T-K hybrids.

### Screening of Hybrid Strains

The hybrid library was spread on YPD agar plates with 50 µg/mL clonNAT, 200 µg/mL zeocin, and 300 µg/mL G418. The plates were incubated at 30°C for 3 days, and then 1,000 colonies were selected. The cells of each colony were suspended in distilled water and inoculated on YPD plates containing 15 mM acetic acid and 15 mM formic acid. The plates were incubated at 38°C for 3 days. Rapidly growing colonies were selected for fermentation tests to determine their fermentation performance of hybrid strains and their parental strains in YPX medium at 38°C with 15 mM acetic acid and 15 mM formic acid.

### Fermentation Conditions

Yeast strains were precultured in 5 mL YPD medium containing 50 µg/mL clonNAT, 200 µg/mL zeocin, and 300 µg/mL G418 at 150 rpm and 30°C for 24 h, followed by aerobic culturing in 500 mL YPD medium with 50 µg/mL clonNAT at 150 rpm and 30°C for 48 h. The cells were then harvested by centrifugation at 3,000 × *g* for 10 min and washed twice with distilled water. Cells were inoculated in 50 mL YPX medium with or without 20 mM acetic acid and 15 mM formic acid in closed bottles equipped with a CO_2_ outlet at an initial cell concentration of 50 g wet cells/L. Temperature was controlled using a water bath equipped with a magnetic stirrer. Fermentation temperatures were set to 30 or 38°C with stirring at 500 rpm. The concentrations of xylose and ethanol in the fermentation medium were determined by high-performance liquid chromatography (Shimadzu, Kyoto, Japan) as previously described (Hasunuma et al., 2011).

### DNA Microarray Analysis

Genome-wide DNA microarray analysis was conducted as previously described (Ismail et al., 2013). Samples obtained after 3 h of fermentation were used for RNA preparation. Scanned data were analyzed using GeneSpring GX ver. 12.6 software (Agilent Technologies). Each microarray sample was analyzed in triplicate. Gene expression was calculated using normalized data and genes with expression differences of twofold or higher were reported. Significant shared gene ontology (GO) terms for biological processes for all significantly expressed genes in the hybrid strain were searched using GO Term Finder (version 0.83) available at the *Saccharomyces* Genome Database (http://www.yeastgenome.org), with a *p*-value cutoff of 0.01. All raw and normalized gene expression data are available in the Gene Expression Omnibus, with the accession number of GSE101786.

### Quantitative Real-time PCR (qRT-PCR)

The isolated total RNA described above was used for qRT-PCR. Reverse transcription and qRT-PCR were conducted as previously described (Ismail et al., 2013). Gene expression levels of target genes were normalized to that of the housekeeping gene *ACT1*. Primers used for qRT-PCR are listed in Table S1 in Supplementary Material.

## Results

### Genome Shuffling and Screening of Hybrid Strains

**Figure 1:**
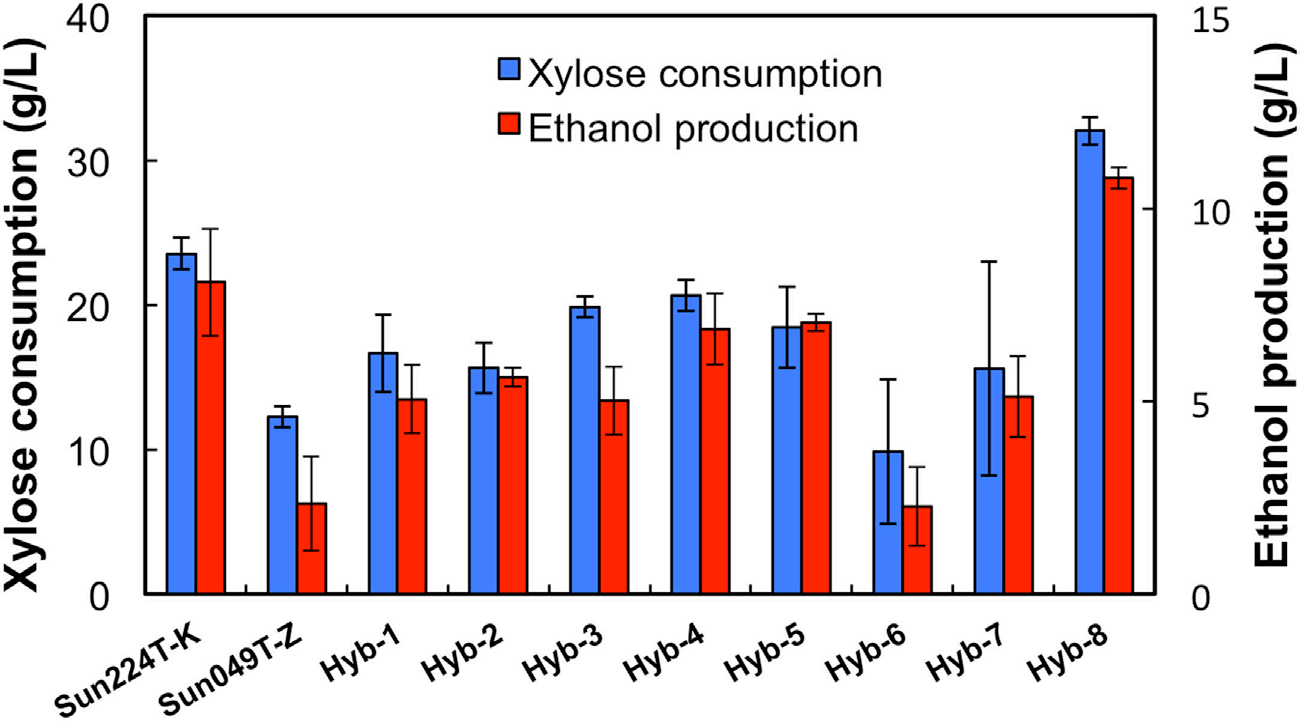
Fermentation performance of hybrid strains and their parental strains at 38°C with 15 mM acetic acid and 15 mM formic acid after 48 h fermentation. Error bars indicate SDs of three independent experiments.

Two xylose-utilizing diploid yeasts with different genetic backgrounds (Sun049T-Z and Sun224T-K) were used as the parental strains for genome shuffling. The mutagenized genome pools derived from these strains were shuffled by sporulation and mating as described in the Section “Materials and Methods.” The obtained library of the shuffled strains was subjected to a two-step screening process to select for a strain possessing tolerances to both high temperature and acids. First, 1,000 colonies were selected from the library, and the growth capacity of cells from each colony was evaluated under heat and acid co-stress conditions. During cultivation, eight fast-growing strains were selected (Hyb-1–8). Next, ethanol fermentation from xylose using the selected hybrid strains and the parental strains was performed under heat and acid co-stress conditions for 48 h. The results are shown in Figure 1. All hybrid strains tested in this study produced ethanol from xylose under co-stress conditions, while fermentation abilities varied by strain. Among the hybrid strains, Hyb-8 exhibited remarkably high xylose consumption and ethanol production (32.0 ± 0.9 and 10.8 ± 0.3 g/L at 48 h, respectively), with higher values than those of the parental strains. The Hyb-8 strain was selected for further characterization as described below.

### Ethanol Fermentation under Different Stress Conditions

**Figure 2:**
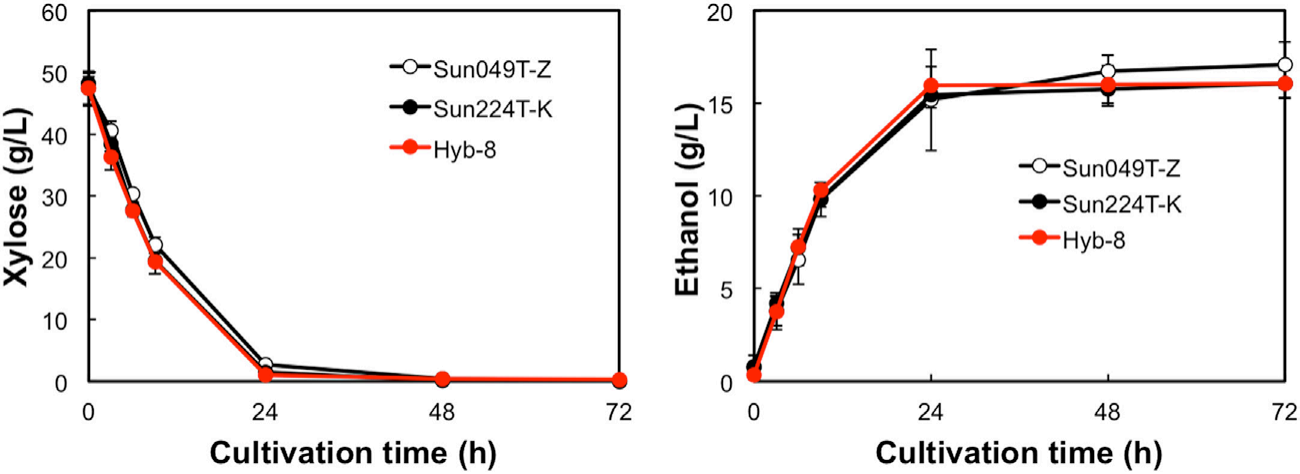
Time course of ethanol production from xylose at 30°C without organic acids. Error bars indicate SDs of three independent experiments.

**Figure 3:**
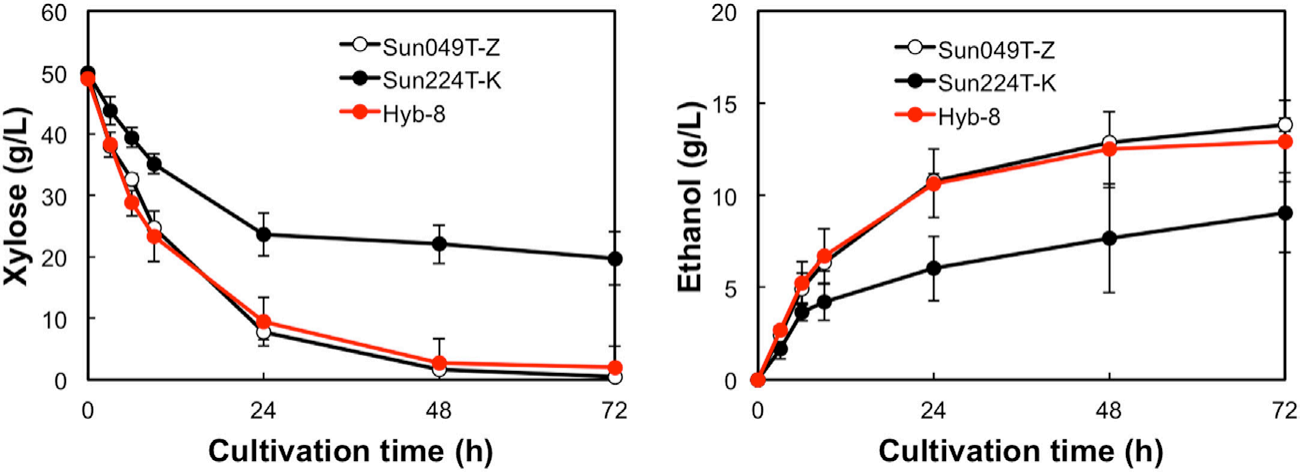
Time course of ethanol production from xylose at 38°C without organic acids. Error bars indicate SDs of three independent experiments.

**Figure 4:**
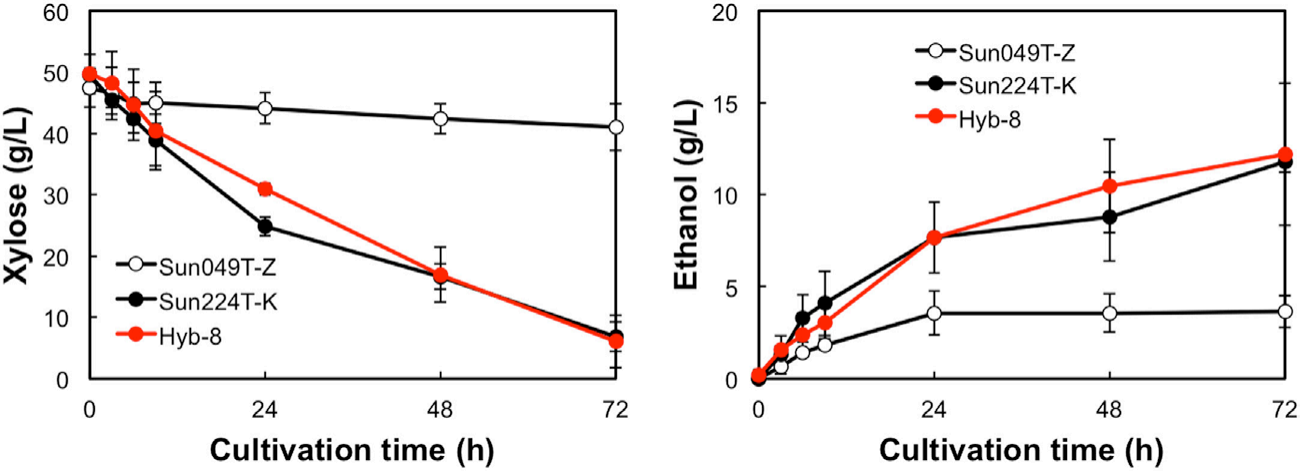
Time course of ethanol production from xylose at 30°C with 20 mM acetic acid and 15 mM formic acid. Error bars indicate SDs of three independent experiments.

**Figure 5:**
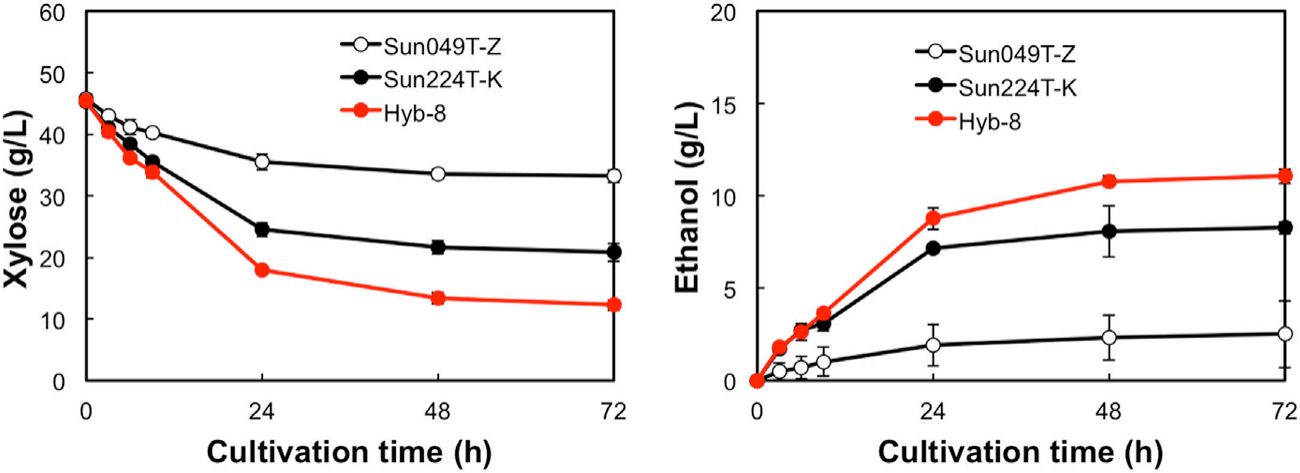
Time course of ethanol production from xylose at 38°C with 20 mM acetic acid and 15 mM formic acid. Error bars indicate SDs of three independent experiments.

The fermentation performances of the selected Hyb-8 strain and the parental strains (Sun049T-Z and Sun224T-K) were evaluated under non-stress, heat stress, acid stress, and heat and acid co-stress conditions. All fermentation tests were performed under microaerobic conditions. The xylose consumptions and ethanol productions of Hyb-8 and its parental strains were similar under non-stress conditions (Figure 2). Under heat stress (Figure 3), Sun049T-Z exhibited higher fermentation performance than Sun224T-K. In contrast, under acid stress (Figure 4), the xylose consumption and ethanol production of Sun049T-Z were significantly inhibited, while Sun224T-K exhibited a relatively high fermentation performance. Compared with the parental strains, the hybrid strain Hyb-8 maintained high fermentation ability under both heat stress conditions and acid stress conditions (Figures 3 and 4). Furthermore, under heat and acid co-stress, Hyb-8 achieved higher xylose consumption and ethanol production (33.1 ± 0.6 and 11.1 ± 0.4 g/L at 72 h, respectively) than those of both parental strains (Figure 5). The ethanol yield of this hybrid strain was approximately 0.33 g/g xylose consumed. Since the theoretical maximum ethanol yield from xylose via the pentose phosphate pathway is 0.511 g/g xylose consumed, the ethanol yield of this strain represents approximately 65% of the theoretical yield.

### DNA Microarray and qRT-PCR Analyses

To screen for key genes involved in the fermentation performance under heat and acid co-stress, a transcriptomic analysis of hybrid strain Hyb-8 and its parental strains (Sun049T-Z and Sun224T-K) was performed. Genes upregulated in Hyb-8 relative to expression levels in the parental strains during the early phase (3 h) of fermentation at 38°C with 20 mM acetic acid and 15 mM formic acid were identified using DNA microarrays. Only genes with expression levels at least twofold higher than those in both parental strains were taken into consideration. The selected genes are listed in Table 2. DNA microarray revealed that 13 genes were upregulated in Hyb-8 compared to levels in both parental strains. Following biological process ontology search using GO Term Finder, we found that 5 of the 13 genes (*TIS11, SIT1, FET3, FTR1*, and *IZH4*) were related to cellular transition metal ion homeostasis (*p*-value = 6.35e−06). Genes responsible for sterol biosynthesis, *ERG25* (C-4 methyl sterol oxidase) and *ERG3* (C-5 sterol desaturase), were also upregulated in Hyb-8. In addition, increased expression of genes encoding endo-polygalacturonase (*PGU1*), phenylpyruvate decarboxylase (*ARO10*), zinc finger protein involved in control of meiosis (*RME1*), master regulator of meiosis (*IME1*), NADPH-dependent quinone reductase (*ZTA1*), and cell wall mannoprotein (*DAN4*) was observed in Hyb-8.

**Table 2:** Upregulated genes in Hyb-8 compared to both Sun049T-Z and Sun224T-K under heat and acid co-stress.

Gene	Synthetic name	Description	Gene ontology term for biological processes	Fold change^a^	
				Vs. Sun049T-Z	Vs. Sun224T-K
*TIS11*	YLR136C	mRNA-binding protein during iron starvation	Cellular transition metal ion homeostasis	14.8	7.8
*SIT1*	YEL065W	Ferrioxamine B transporter	Cellular transition metal ion homeostasis	11.5	3.9
*FET3*	YMR058W	Ferro-O_2_-oxidoreductase	Cellular transition metal ion homeostasis	9.9	4.7
*FTR1*	YER145C	High affinity iron permease	Cellular transition metal ion homeostasis	5.1	2.6
*IZH4*	YOL101C	Membrane protein involved in zinc ion homeostasis	Cellular transition metal ion homeostasis	3.7	2.5
*PGU1*	YJR153W	Endo-polygalacturonase		15.0	2.6
*ERG25*	YGR060W	C-4 methyl sterol oxidase		10.6	2.6
*ERG3*	YLR056W	C-5 sterol desaturase		4.9	2.1
*ARO10*	YDR380W	Phenylpyruvate decarboxylase		4.6	2.2
*RME1*	YGR044C	Zinc finger protein involved in control of meiosis		3.3	2.1
*IME1*	YJR094C	Master regulator of meiosis		2.6	2.6
*ZTA1*	YBR046C	NADPH-dependent quinone reductase		2.2	2.4
*DAN4*	YJR151C	Cell wall mannoprotein		2.1	2.1

*^a^All fold changes were significant with p-value < 0.0.5*.

To validate the microarray data, transcript levels of genes associated with metal ion homeostasis (*TIS11, SIT1, FET3, FTR1*, and *IZH4*) and sterol biosynthesis (*ERG25* and *ERG3*) in Hyb-8, and the parental strains were analyzed by qRT-PCR. Although only the expression level of *IZH4* in Hyb-8 was slightly lower than that in Sun224T-K in the qRT-PCR analysis, all the other selected genes were upregulated in Hyb-8 compared to the levels in both parental strains as in the microarray analysis (Table S2 in Supplementary Material).

## Discussion

Xylose-utilizing yeasts with multiple tolerances to high temperature and fermentation inhibitors should be developed for cost-effective SSCF of lignocellulosic materials. In this study, we shuffled the mutagenized genome pools derived from two xylose-assimilating diploid yeasts with different genetic backgrounds (Sun049T-Z and Sun224T-K) by sporulation and mating and isolated the hybrid xylose-assimilating *S. cerevisiae* strain Hyb-8. In ethanol fermentation from 50 g/L xylose, the parental strains Sun049T-Z and Sun224T-K exhibited relatively high fermentation performances under heat stress conditions and acid stress conditions, respectively, while the fermentation performances of these strains were significantly inhibited under the opposing conditions. In contrast, the hybrid strain Hyb-8 maintained high fermentation ability under both these conditions. Furthermore, under heat and acid co-stress, Hyb-8 achieved higher xylose consumption and ethanol production (33.1 ± 0.6 and 11.1 ± 0.4 g/L at 72 h, respectively) than those of both parental strains (Figure 5). The ethanol yield of this hybrid strain was approximately 0.33 g/g xylose consumed. These results suggest that Hyb-8 inherited desirable traits from both parental strains and demonstrates strong potential for cost-effective SSCF of lignocellulosic materials.

Recently, many researchers have reported the development of yeasts with tolerances against a single harsh condition using the genome shuffling technique (Steensels et al., 2014). However, because of the genetic complexity of these tolerances, the development of robust yeasts possessing tolerances to both high temperature and acids has only been reported in a few cases (Zheng et al., 2011b; Lu et al., 2012). Moreover, to our knowledge, the application of genome shuffling to improve the fermentation performance of xylose-assimilating *S. cerevisiae* under multiple stress conditions has not been reported.

We also performed genome-wide DNA microarray analysis of Hyb-8 and its parental strains to screen for genes that might be important in ethanol fermentation under heat and acid co-stress. Interestingly, the following five genes associated with cellular transition metal ion homeostasis were found to be significantly up-regulated in Hyb-8: mRNA-binding protein during iron starvation (*TIS11*), ferrioxamine B transporter (*SIT1*), ferro-O_2_-oxidoreductase (*FET3*), high-affinity iron permease (*FTR1*), and membrane protein involved in zinc ion homeostasis (*IZH4*). The increased expression of these genes was also indicated in qRT-PCR analysis except for *IZH4*. Although the relationships between these genes and tolerances to fermentation inhibitors and high temperature have not been reported, this result suggests that intracellular pools of metal ions are involved in the multiple stress tolerances of Hyb-8. Ismail et al. (2014) reported that supplementation of three metal ions (Zn^2+^, Mg^2+^, and Ca^2+^) increased the tolerance of *S. cerevisiae* toward acetic acid stress. In addition, Bellí et al. (2004) reported that a group of genes implicated in metal ion homeostasis, including *TIS11, SIT1*, and *FTR1*, was induced in Δ*grx5* cells, which are models for studying the influence of continuous oxidative stress on gene expression. During fermentation, yeast cells are exposed to oxidative stress caused by reactive oxygen species, which are respiratory byproducts that damage various cellular components (Hori et al., 2009). Recently, Lertwattanasakul et al. (2015) reported the transcriptomic analysis of the thermotolerant and xylose-fermenting yeast *Kluyveromyces marxianus* DMKU 3-1042 under different growth conditions and indicated that oxidative stress response genes were highly induced under both high temperature (45°C) and xylose-utilizing conditions compared to levels under glucose-utilizing conditions at 30°C. Based on their results, they speculated that oxidative stress increases and accumulates under high temperature and xylose-utilizing conditions in yeast. The increased expression of the genes described above in Hyb-8 may play a crucial role in maintaining its high xylose fermentation ability under multiple stress conditions by reducing oxidative stress. Hyb-8 also exhibited enhanced expression of *ZTA1*, encoding NADPH-dependent quinone reductase, and this gene has also been suggested to be associated with the oxidative stress response in *S. cerevisiae* (Fernández et al., 2007).

Hyb-8 also exhibited elevated gene expression in the ergosterol biosynthesis pathway, i.e., *ERG25* (C-4 methyl sterol oxidase) and *ERG3* (C-5 sterol desaturase) in both DNA microarray and qRT-PCR analyses. Ergosterol is the major sterol in the yeast cell membrane, and the ratio, composition, and structure of sterols affect the fluidity of membranes (Dufourc, 2008; Caspeta et al., 2014). Vanegas et al. (2012) reported that increased ergosterol content strengthens the membrane structure and could improve ethanol tolerance in yeast. Caspeta et al. (2014) reported the increased expression of genes involved in ergosterol biosynthesis in thermally adapted *S. cerevisiae* strains. Sterol biosynthesis is an oxygen-requiring pathway, and anaerobically grown yeast cells are auxotrophic for ergosterol (Parks and Casey, 1995). Although sterol composition analyses of Hyb-8 and its parental strains are needed, the enhanced expression of *ERG25* and *ERG3* may contribute to the high fermentation ability of Hyb-8 by maintaining cell membrane homeostasis under microaerobic and multiple stress conditions.

The increased expression of *ARO10*, encoding phenylpyruvate decarboxylase, which is involved in amino acid metabolism, was also observed in Hyb-8. This enzyme is reported to play a major role in aromatic amino acid catabolism *in vivo* (Kneen et al., 2011). These amino acids are important for organic acid tolerance because the transport of these amino acids may be inhibited in the presence of organic acids (Bauer et al., 2003). Li and Yuan (2010) reported that several genes involved in tryptophan metabolism in *S. cerevisiae* were upregulated in response to acetic acid addition.

In the present study, genes encoding endo-polygalacturonase (*PGU1*), zinc finger protein involved in control of meiosis (*RME1*), master regulator of meiosis (*IME1*), and cell wall mannoprotein (*DAN4*) were also upregulated in Hyb-8. Although the individual roles of these genes are unclear, their enhanced expression may play a role in the multiple stress tolerances and/or xylose fermentation ability of Hyb-8. For instance, Satomura et al. (2013) reported that *RME1* was upregulated in the thermally adapted *S. cerevisiae* strain YK60-1 compared to levels in its parental strain, MT8-1.

In this study, we developed a novel xylose-utilizing *S. cerevisiae* strain with improved fermentation ability under heat and acid co-stress using the genome shuffling technique. The shuffled strain Hyb-8 successfully inherited desirable traits from both parental strains and showed high xylose fermentation ability under heat and acid co-stress. Although further investigations are necessary, Hyb-8 has strong potential for cost-effective SSCF of lignocellulosic materials. Moreover, genome-wide DNA microarray analysis revealed the unique transcriptomic profile of Hyb-8 under co-stress conditions. The transcriptome data derived from this study will be useful in understanding the mechanisms of multiple tolerances to high temperature and acids in yeast and facilitate the development of robust yeast strains for SSCF.

## Author Contributions

KI involved in the study design and wrote the manuscript. RI carried out genome shuffling, fermentation, and microarray experiments. TB trained RI to conduct microarray analysis and performed statistical analysis of the data. TH participated in the design of the study and corrected the manuscript. AK conceived and designed the study and corrected the manuscript. All authors read and approved the manuscript.

## Conflict of Interest Statement

The authors declare that the research was conducted in the absence of any commercial or financial relationships that could be construed as a potential conflict of interest.

## Abbreviations

GEO, gene expression omnibus; GO, gene ontology; HPLC, high-performance liquid chromatography; OD_600_, optical density at 600 nm; qRT-PCR, quantitative real-time PCR; SSCF, simultaneous saccharification and co-fermentation; UV, ultraviolet; YPD, yeast extract peptone dextrose; YPX, yeast extract peptone xylose.
