# Supplementary material for: Improvement of Xylose Fermentation Ability under Heat and Acid Co-Stress in Saccharomyces cerevisiae Using Genome Shuffling Technique
Source: Front Bioeng Biotechnol. 2017 Dec 20;5:81. doi: 10.3389/fbioe.2017.00081 (PMC5742482; doi:10.3389/fbioe.2017.00081)
Supplement: Supplementary file 1 [file Table_1.DOCX]

Supplementary Material

Improvement of xylose fermentation ability under heat and acid co-stress in *Saccharomyces cerevisiae* using genome shuffling technique

Kentaro Inokuma, Ryo Iwamoto, Takahiro Bamba, Tomohisa Hasunuma, Akihiko Kondo^*^

*** Correspondence:** Akihiko Kondo: akondo@kobe-u.ac.jp

# Supplementary Table S1

Primers used in qRT-PCR.

| Name | Sequence |
| --- | --- |
| *TIS11*-RT-F | GGTGAAAAGCCAAGTGCAGG |
| *TIS11*-RT-R | GTCCGTGAGCAAACTGACAC |
| *STI1*-RT-F | CGGCTGTAGGCCAGATCTTC |
| *STI1*-RT-R | CGCTTGCGACTCAATGATGG |
| *FET3*-RT-F | GAAGTCCAGCCCGATACGAC |
| *FET3*-RT-R | TACCGTCGATTTCGACCACG |
| *FTR1*-RT-F | TGATCTCCGCCGGTCTTTTC |
| *FTR1*-RT-R | ATTACCATCACCGCCTTCGG |
| *IZH4*-RT-F | AGTGTGTTGTTCCTTGCCAC |
| *IZH4*-RT-R | ACCAAAGCCACAAGTTCCCA |
| *ERG25*-RT-F | GCGCTGAACACCACGATTTG |
| *ERG25*-RT-R | GGAGGCCTTAGCTTCTGGAC |
| *ERG3*-RT-F | TCGCTATTGCCTCGTTCCAG |
| *ERG3*-RT-R | CACGTAGCTGAGACTAGCCG |
| *ACT1*-RT-F | TGGATTCCGGTGATGGTGTT |
| *ACT1*-RT-R | TCAAAATGGCGTGAGGTAGAGA |
