# Supplementary material for: Improvement of Xylose Fermentation Ability under Heat and Acid Co-Stress in Saccharomyces cerevisiae Using Genome Shuffling Technique
Source: Front Bioeng Biotechnol. 2017 Dec 20;5:81. doi: 10.3389/fbioe.2017.00081 (PMC5742482; doi:10.3389/fbioe.2017.00081)
Supplement: Supplementary file 2 [file Table_2.DOCX]

Supplementary Material

Improvement of xylose fermentation ability under heat and acid co-stress in *Saccharomyces cerevisiae* using genome shuffling technique

Kentaro Inokuma, Ryo Iwamoto, Takahiro Bamba, Tomohisa Hasunuma, Akihiko Kondo^*^

*** Correspondence:** Akihiko Kondo: akondo@kobe-u.ac.jp

# Supplementary Table S2

Comparison of fold change in gene expression levels in Hyb-8 between DNA microarray and qRT-PCR analyses

| Gene | Fold change in DNA microarray | |  | Fold change in qRT-PCR | |
| --- | --- | --- | --- | --- | --- |
|  | Vs. Sun049T-Z | Vs. Sun224T-K |  | Vs. Sun049T-Z | Vs. Sun224T-K |
| *TIS11* | 14.8 (1.23e-08)^a^ | 7.8 (1.55e-05) |  | 8.2 (4.86e-05) | 10.3 (4.33e-05) |
| *SIT1* | 11.5 (7.05e-08) | 3.9 (7.67e-07) |  | 2.5 (1.03e-05) | 2.8 (1.52e-06) |
| *FET3* | 9.9 (6.44e-07) | 4.7 (5.43e-06) |  | 1.1 (2.03e-02) | 3.6 (1.25e-05) |
| *FTR1* | 5.1 (9.91e-07) | 2.6 (1.76e-05) |  | 1.2 (6.31e-03) | 2.4 (9.71e-05) |
| *IZH4* | 3.7 (1.66e-06) | 2.5 (2.85e-04) |  | 1.6 (2.05e-03) | 0.7 (3.35e-03) |
| *ERG25* | 10.6 (1.78e-07) | 2.6 (1.08e-03) |  | 1.8 (3.78e-05) | 1.9 (1.50e-04) |
| *ERG3* | 4.9 (5.84e-06) | 2.1 (1.77e-02) |  | 2.0 (4.16e-04) | 1.5 (4.03e-04) |

^a^*p*-values are shown in parentheses
